# Sex Difference in Corticosterone-Induced Insulin Resistance in Mice

**DOI:** 10.1210/en.2019-00194

**Published:** 2019-07-02

**Authors:** Kasiphak Kaikaew, Jacobie Steenbergen, Theo H van Dijk, Aldo Grefhorst, Jenny A Visser

**Affiliations:** 1 Department of Internal Medicine, Erasmus MC, University Medical Center Rotterdam, Rotterdam, Netherlands; 2 Department of Physiology, Faculty of Medicine, Chulalongkorn University, Bangkok, Thailand; 3 Department of Laboratory Medicine, University Medical Center Groningen, Groningen, Netherlands; 4 Department of Experimental Vascular Medicine, Amsterdam University Medical Centers, Location AMC, Amsterdam, Netherlands

## Abstract

Prolonged exposure to glucocorticoids (GCs) causes various metabolic derangements. These include obesity and insulin resistance, as inhibiting glucose utilization in adipose tissues is a major function of GCs. Although adipose tissue distribution and glucose homeostasis are sex-dependently regulated, it has not been evaluated whether GCs affect glucose metabolism and adipose tissue functions in a sex-dependent manner. In this study, high-dose corticosterone (rodent GC) treatment in C57BL/6J mice resulted in nonfasting hyperglycemia in male mice only, whereas both sexes displayed hyperinsulinemia with normal fasting glucose levels, indicative of insulin resistance. Metabolic testing using stable isotope-labeled glucose techniques revealed a sex-specific corticosterone-driven glucose intolerance. Corticosterone treatment increased adipose tissue mass in both sexes, which was reflected by elevated serum leptin levels. However, female mice showed more metabolically protective adaptations of adipose tissues than did male mice, demonstrated by higher serum total and high-molecular-weight adiponectin levels, more hyperplastic morphological changes, and a stronger increase in mRNA expression of adipogenic differentiation markers. Subsequently, *in vitro* studies in 3T3-L1 (white) and T37i (brown) adipocytes suggest that the increased leptin and adiponectin levels were mainly driven by the elevated insulin levels. In summary, this study demonstrates that GC-induced insulin resistance is more severe in male mice than in female mice, which can be partially explained by a sex-dependent adaptation of adipose tissues.

Glucocorticoids (GCs) are steroid hormones produced by the adrenal gland. External stressors such as infection, trauma, food deprivation, and physical or psychological stress enhance the activity of the hypothalamic-pituitary-adrenal (HPA) axis and trigger the adrenal gland to secrete an endogenous GC: cortisol for humans or corticosterone for rodents (1).

Exogenous synthetic GCs are widely prescribed for a number of autoimmune diseases and allergic reactions due to their immunosuppressive properties (2). However, prolonged exposure to elevated endogenous GCs, such as in Cushing syndrome, or to exogenous GCs leads to various metabolic derangements, such as progressive weight gain, truncal obesity due to expansion of the visceral white adipose tissue (WAT), loss of subcutaneous WAT mass, and development of insulin resistance that might result in diabetes mellitus (2–5). These effects are largely attributable to the role of GC in the control of glucose homeostasis, as they promote hepatic glucose production (gluconeogenesis) and inhibit glucose utilization in WAT and skeletal muscles (1, 4).

The HPA axis is controlled in a sexually dimorphic manner: female rodents have higher basal and stress-induced corticosterone levels with a less robust negative feedback on the HPA axis than do males (6, 7). The fact that the concentrations of corticosteroid-binding globulin, the glycoprotein that binds 80% of circulating corticosterone, are higher in females than in males likely contributes to this sex difference (6, 8). Sex steroid hormones are also involved in the sex-dependent control of the HPA axis (6, 7). In adult male rats, castration and estradiol treatment increase responsiveness of the HPA axis to external stressors whereas ovariectomy and androgen replacement in female rats decrease this response (7).

Intriguingly, adipose tissue distribution and function show many sex-dependent characteristics as well. Concerning WAT distribution, females have relatively more subcutaneous WAT and less visceral WAT than do males (9, 10). Females also have a relatively higher activity of the metabolically active brown adipose tissue (BAT) and have more brown-like adipocytes in their WAT depots (11, 12). Furthermore, glucose metabolism has been shown to differ between males and females, with female mice being more insulin sensitive and glucose tolerant than male mice (13).

Despite these sex differences in HPA axis regulation, adipose tissue distribution, and glucose homeostasis, high-dose GC- or stress-induced adverse effects have only been studied in male rodents (14, 15). Whether the metabolic consequences of exposure to high-dose GC differ between males and females is still unknown. To address this knowledge gap, we have studied the effects of 2-week high-dose corticosterone on whole-body glucose metabolism and adipose tissue function in both male and female C57BL/6J mice.

## Materials and Methods

### Animals, housing conditions, and corticosterone treatment

Eight-week-old C57BL/6J mice (24 males and 24 females) were obtained from Charles River Laboratories (Maastricht, Netherlands). Upon arrival, mice were group housed (three mice per cage) at room temperature (RT; ∼22°C) on a 12-hour light/12-hour dark cycle (lights on at 8:00 am). Chow food pellets [801722 CRM (P), Special Diets Services, Essex, UK] and water were available *ad libitum*. We provided tissue papers (Tork extra soft facial tissue, SCA Hygiene Products, Zeist, Netherlands) as nesting material and woodchips (Lignocel BK 8/15, J. Rettenmaier & Söhne, Rosenberg, Germany) as bedding material. All experimental procedures were approved by the Animal Ethics Committee at Erasmus MC, Rotterdam, Netherlands.

After 10 to 14 days of acclimatization, a corticosterone pellet [50 mg of corticosterone (Sigma-Aldrich, Zwijndrecht, Netherlands) and 50 mg of cholesterol] or a vehicle pellet (100 mg of cholesterol) was implanted subcutaneously at the dorsal region of the neck under isoflurane (Teva Pharmachemie, Haarlem, Netherlands) anesthesia and carprofen (Rimadyl Cattle, Pfizer Animal Health, Capelle aan den IJssel, Netherlands) analgesia. Subsequently, mice were single housed and enrolled in experiment 1 or 2.

### Experiment 1: nonfasting glucose monitoring and endpoint blood and tissue collection

For experiment 1, mice were weighed on day 0 (before pellet implantation) and on days 3, 5, 7, 10, and 12 after pellet implantation at 1:00 pm. At the same time, their nonfasting blood glucose (NFBG) level was determined by tail-tip bleeding using a glucometer and test strips (FreeStyle Freedom Lite, Abbott, Hoofddorp, Netherlands). On day 12, we weighed the food pellets and transferred the mice to a new cage with similar conditions. On day 14 at 8:00 am, the mice and food pellets were weighed again, the NFBG was measured, and the bedding material was collected for further processing. Next, we transferred the mice to clean cages with similar conditions except for the presence of food pellets. At 1:00 pm (after 5 hours of fasting), the fasting blood glucose (FBG) level was determined and the mice were euthanized by cardiac puncture under isoflurane anesthesia. Thymus involution was confirmed in the corticosterone-treated mice. Blood was stored immediately at 4°C and various tissues (*e.g.*, BAT, WAT, quadriceps femoris muscle, and liver) were dissected, weighed, and snap-frozen in liquid nitrogen or fixed in 4% paraformaldehyde (PFA) in PBS at RT. The inguinal (also known as posterior subcutaneous) WAT (iWAT) and gonadal WAT (gWAT) depots were cut in half. One half of iWAT was snap-frozen; one half of gWAT was further divided and snap-frozen or fixed as described above. The other half of iWAT and gWAT was washed in PBS and preincubated in DMEM/F12 medium (catalog no. 21331020, Gibco, Life Technologies Europe, Bleiswijk, Netherlands) with 2% fatty acid–free BSA (FF-BSA; catalog no. 03117057001, Roche Diagnostics, Mannheim, Germany) at RT for subsequent *ex vivo* stimulation.

Serum (obtained after an overnight clotting at 4°C) and snap-frozen tissues were stored at −80°C until analyses. After 24-hour fixation in PFA at RT, tissues were stored in 70% ethanol until histological analysis. Feces were collected from the bedding material, air-dried, weighed, crushed, and extracted with ethanol for fecal corticosterone measurement, as described previously (16).

### Experiment 2: IP glucose tolerance test

For experiment 2, mice were weighed and their NFBG was determined on days 0 (before pellet implantation), 7, and 14 at 8:00 am. Next, food pellets were removed and the mice were fasted until 1:00 pm when their FBG was determined and one blood spot (∼6 µL) was collected on filter paper (TFN 180 g/m^2^, Sartorius Stedim Biotech, Göttingen, Germany) for fasting blood insulin (FBI) measurement. Additionally, on day 14 mice were subjected to an IP glucose tolerance test (IPGTT). For this, the mice received an IP injection of 2 g/kg glucose [20% glucose solution, which contains 95% d-(+)-glucose (Sigma-Aldrich) and 5% [U-^13^C_6_]-d-glucose (Cambridge Isotope Laboratories, Andover, MA)]. Blood glucose levels were determined and two blood spots (∼3 µL for glucose kinetic analysis and ∼6 µL for insulin measurement) were collected on filter paper at 5, 15, 30, 45, 75, and 120 minutes after glucose injection. After the experiment, mice were euthanized by cervical dislocation under isoflurane anesthesia. Blood spots were air-dried for 2 hours and stored at −20°C (for insulin measurement) or at RT (for glucose extraction).

### Insulin stimulation of WAT explants

Pieces of iWAT and gWAT preincubated with 2% FF-BSA in DMEM/F12 (from experiment 1) were cut into small pieces of ∼20 mg and incubated in DMEM/F12 medium containing 2% FF-BSA (Roche Diagnostics) with or without 1 µM insulin (Sigma-Aldrich) at 37°C in a humidified incubator with 5% CO_2_ for 2 hours (refreshed once with fresh solution after 1 hour of incubation). Subsequently, tissues were washed twice in cold PBS and stored at −80°C until protein extraction.

### Adipose tissue histology and adipocyte size quantification

PFA-fixed gWAT, anterior subcutaneous (also known as axillary) WAT (aWAT), and BAT were embedded in paraffin. After manually sectioned with a microtome, 8-µm-thick WAT and 5-µm-thick BAT sections were mounted on glass slides and stained with hematoxylin and eosin.

Representative images from three random sections of gWAT and aWAT from each animal were taken with a digital imaging system (Nikon Eclipse E400 and Nikon Digital Sight DS-L1, Nikon Corporation, Tokyo, Japan). To quantify adipocyte size, we used an automated mode of Adiposoft, a plug-in of Fiji (advanced distribution of ImageJ) software for accurately analyzing number and size of adipocytes (17).

### Circulating hormone and adipokine quantification by ELISA

Serum and fecal concentrations of hormones and adipokines of the mice from experiment 1 were determined according to the manufacturers’ protocols. Serum total and high-molecular-weight (HMW) adiponectin levels were measured with a mouse HMW and total adiponectin ELISA kit (18). Serum leptin was measured with a mouse/rat leptin ELISA kit (19). Serum and fecal corticosterone levels were determined with a corticosterone ELISA kit (20).

The insulin concentrations in the blood spots collected in experiment 2 were determined as previously described (21) using a rat insulin ELISA kit (22) with the Mouse Insulin Standard (90020, Crystal Chem, Zaandam, Netherlands). In brief, a completely filled blood spot on filter papers (6-mm diameter) was punched out and eluted in guinea pig anti-insulin in sample diluent overnight at 4°C, followed by the standard procedure of the ELISA kit.

### Derivatization and gas chromatography–mass spectrometry measurements of glucose

Extraction of glucose from the filter paper blood spots, derivatization of the extracted glucose, and gas chromatography–mass spectrometry (GC-MS) measurements of the glucose derivatives were done according to the analytical procedure described before (23). In short, a disk was punched out of the blood spots, glucose was extracted from the disk by incubating it in ethanol/water (10:1 v/v), and glucose was derivatized to its pentaacetate ester. Samples were analyzed by GC-MS (Agilent 5975C inert MSD, Agilent Technologies, Amstelveen, Netherlands) with separation of derivatives on 30-m × 0.25-mm interior diameter (0.25-µm film thickness) capillary columns (ZB-1701, Phenomenex, Utrecht, Netherlands) and with positive-ion chemical ionization with methane. Measured by GC-MS, the fractional isotopomer distribution (M_0_ to M_6_) was corrected for the fractional distribution due to natural abundance of ^13^C by multiple linear regression as described by Lee *et al.* (24) to obtain the excess fractional distribution of mass isotopomers (M_0_ to M_6_) due to the dilution of administered [U-_13_C_6_]-d-glucose; that is, M_6_ represents the fractional contribution of the administered tracer and was used in the calculations of blood glucose kinetics.

### Calculation of blood glucose metabolism

**Figure 1.**
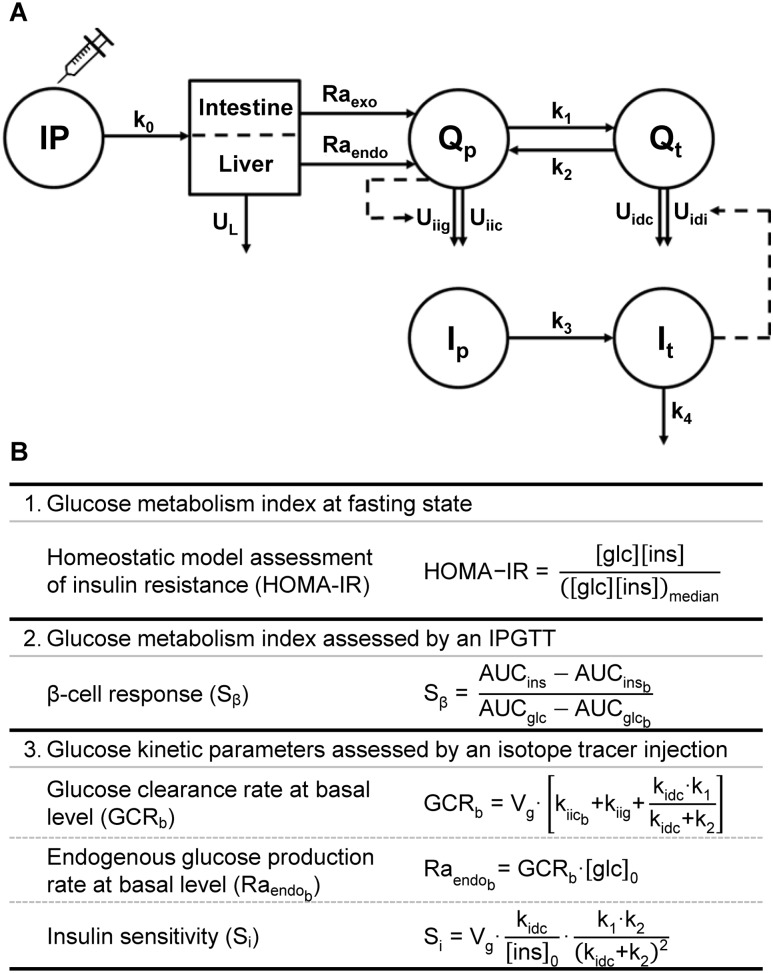
Compartmental model and formulas used for calculating blood glucose kinetics. (A) Kinetic model used to calculate the kinetic parameters of glucose metabolism upon an IPGTT in mice. Upon injection into the IP compartment, the injected glucose passes the liver to contribute to the accessible plasma glucose pool (Q_p_) and to the inaccessible “tissue” glucose pool (Q_t_). Additionally, glucose produced/released by tissues such as the intestine and liver also contribute to Q_p_. As such, two rates of appearances can be distinguished, namely that of exogenous injected glucose (Ra_exo_) and of endogenous glucose (Ra_endo_). The Q_p_ is in equilibrium with Q_t_ via two rate constants (k_1_ and k_2_). Disposal of glucose (U) from the Q_p_ can be divided in insulin-independent glucose-dependent disposal (U_iig_) and insulin-independent constant disposal (U_iic_). Disposal from the Q_t_ is the sum of insulin-dependent constant disposal (U_idc_) and insulin-dependent disposal (U_idi_). As with glucose, this model also presumes two compartments for insulin, namely the accessible plasma insulin pool (I_p_) and the inaccessible tissue insulin pool (I_t_). (B) Formulas for assessing glucose metabolism indexes and kinetic parameters. HOMA-IR, an acceptable surrogate index for insulin resistance when applying a mouse-specific constant (26, 27), was calculated relative to a median of the vehicle-treated male mice. *β*-Cell response was estimated as changes in plasma insulin levels relative to changes in glucose levels (28). Subscript b refers to a basal level, subscript 0 refers to an estimated value at time point 0, and Vg indicates the volume of the accessible pool.

Tracer concentrations were calculated as the product of the blood glucose concentration and the fractional contribution of the tracer at that time point (*t*): [^13^C_6_-glucose]_*t*_ = (M_6_)_*t*_⋅[glucose]_*t*_. To determine the effects of the IPGTT on glucose metabolism in mice, we used an adapted minimal model for glucose metabolism after an oral glucose tolerance test (25). This adapted model is presented in Fig. 1A and was used in SAAM II software (version 2.3, The Epsilon Group, Charlottesville, VA). To generate sufficient input data for this model, measured data of blood glucose, blood insulin, and tracer concentrations were fitted to the following formula to calculate the concentration (*C*) of these metabolites at multiple time points (*t*): $C_{t}\;=C_{b}+C_{\left(1\right)0}\cdot e^{-k_{\left(1\right)}t}\;+\;C_{\left(2\right)0}\cdot e^{-k_{\left(2\right)}t}\;-\;C_{\left(3\right)0}\cdot e^{-k_{\left(3\right)}t}$, where *b* indicates the basal value and 0 indicates the estimated value at time point 0. The bioavailability (*F*) of the bolus was estimated from the tracer curve as follows:

$$F\;=\;1\;-\;\left[\frac{C_{\left(3\right)0}k_{\left(1\right)}k_{\left(2\right)}}{C_{\left(1\right)0}k_{\left(2\right)}k_{\left(3\right)}\;+\;C_{\left(2\right)0}k_{\left(1\right)}k_{\left(3\right)}}\right].$$

The glucose kinetic parameters were calculated using the compartmental model and the relevant equations (26–28), as presented in Fig. 1. In this model, k_1_ and k_2_ are rate constants for the glucose flux from the accessible plasma pool to the inaccessible “tissue” pool and *vice versa*, whereas k_3_ is the rate constant for the insulin flux from the accessible plasma pool to the inaccessible tissue pool, and they are different from the rate constants *k*_(1)_, *k*_(2)_, and *k*_(3)_ used to describe the plasma glucose vs time curve above. We adapted the volume of the accessible glucose pool from literature; that is, 150 mL/kg was suggested by Tissot *et al.* (29) and Gastaldelli *et al.* (30) for humans. The insulin-independent glucose utilization was set to three times the insulin-dependent glucose utilization under basal conditions as was also used for humans (31, 32). Furthermore, under basal conditions the independent utilization flux was estimated at 45% of the endogenous glucose production (EGP). The fractional turnover rates (k_0_ through k_4_) were estimated within the model.

### *In vitro* adipocyte culture

The white preadipocyte cell line 3T3-L1 (33) and the brown preadipocyte cell line T37i [(34); a gift provided by Dr. M. Lombès, Inserm U1185, France] were used to study direct effects of corticosterone and/or insulin on adipocytes. 3T3-L1 preadipocytes were cultured with basal medium [3T3-BM: DMEM 4.5 g/L glucose with l-glutamine and 25 mM HEPES (21063029, Gibco) supplemented with 10% fetal bovine serum (FBS; Gibco) and 100 IU/mL penicillin/100 µg/mL streptomycin (P/S; Gibco)]. Two days after reaching full confluence (differentiation day 0), 3T3-L1 cells were differentiated with differentiation medium 1: 3T3-BM containing 0.5 mM 3-isobutyl-1-methylxanthine, 0.25 µM dexamethasone, and 1 µg/mL insulin (all from Sigma-Aldrich). Starting from day 4, cells were maintained in differentiation medium 2 (3T3-BM containing 1 µg/mL insulin) that was refreshed every 2 to 3 days until full differentiation on day 12. T37i preadipocytes were cultured with basal medium [T37i-BM: DMEM/F12 with l-glutamine (21041025, Gibco) supplemented with 10% FBS, P/S, and 20 mM HEPES (Gibco)]. Two days after reaching full confluence (differentiation day 0), T37i cells were differentiated by adding 2 nM triiodothyronine and 20 nM insulin (both from Sigma-Aldrich) to T37i-BM. This medium was refreshed every 2 to 3 days until full differentiation on day 9.

Before corticosterone and insulin stimulation, the differentiating cells were steroid-starved for 24 hours by replacing FBS with dextran-coated charcoal-treated FBS [prepared by incubating 100 mL of FBS twice with 0.1 g of dextran T250 (Pharmacia, Uppsala, Sweden) and 1 g of activated charcoal (C5510, Sigma-Aldrich) for 30 minutes, centrifuged, and sterile filtered]. Three hours before stimulation, the differentiated cells were starved in starvation medium: DMEM (for 3T3-L1) or DMEM/F12 with 20 mM HEPES (for T37i) supplemented with P/S and 0.2% dextran-coated charcoal-treated FBS. Subsequently, the cells were stimulated for 24 hours in starvation medium containing 1 µL/mL ethanol vehicle control, 1 µM corticosterone, 0.2 µM insulin, or 1 µM corticosterone and 0.2 µM insulin (both from Sigma-Aldrich). After stimulation, the cells were used to determine their glucose uptake or immediately stored at −80°C until RNA isolation or protein extraction. In the latter case, cultured media were also collected, centrifuged, and stored at −20°C for adipokine measurement by ELISA.

### Radioactive glucose uptake

For the glucose uptake studies, the 24-hour corticosterone- and/or insulin-treated cells were washed with PBS and stimulated with 0, 20, or 100 nM insulin in 0.1% FF-BSA (03117057001, Roche Diagnostics) in PBS for 15 minutes. Next, 0.05 µCi of 2-[1-^14^C]-deoxy-d-glucose (PerkinElmer, Waltham, MA) in 0.1% FF-BSA was added to the medium. After an additional 5-minute incubation, the cells were washed twice with cold PBS, lysed with 0.2% SDS solution (Merck, Hohenbrunn, Germany), and protein content in cell lysates was quantified using Advanced protein assay reagent (Cytoskeleton, Denver, CO). Cell lysates were transferred to a scintillation glass vial and homogenized in a scintillation cocktail (Optiphase HiSafe 3, PerkinElmer Health Sciences, Groningen, Netherlands). Radioactivity was detected with a liquid scintillation analyzer (Tri-Carb 2910TR, Packard, PerkinElmer) and reported in counts per minute normalized to protein content.

### Gene expression analysis

RNA was isolated from mouse tissues and cultured cells using the TriPure isolation reagent (Roche Diagnostics) according to the manufacturer’s instructions. Contaminating genomic DNA was removed using RQ1 RNase-free DNase (Promega Corporation, Madison, WI). Purified RNA was quantified with a NanoDrop 8000 spectrophotometer (Thermo Fisher Scientific, Wilmington, DE). Reverse transcription was performed using the Transcriptor high-fidelity cDNA synthesis kit (Roche Diagnostics). Quantitative PCR was performed using FastStart Universal SYBR Green Master (Rox) (Roche Diagnostics) with a QuantStudio 7 flex real-time PCR system (Applied Biosystems, Life Technologies, Carlsbad, CA). Expression of the tested genes was normalized to the indicated housekeeping genes using the 2^−ΔΔCT^ method. Sequences of the primers for all genes are listed in Table 1.

**Table 1.** Primer Sequences

Gene	Forward (5′→3′)	Reverse (5′→3′)
*Adamts1*	TGCTCCAAGACATGCGGCTCAG	TGGTACTGGCTGGCTTCACTTCC
*Adipoq*	GCACTGGCAAGTTCTACTGCAA	GTAGGTGAAGAGAACGGCCTTGT
*Cebpb*	ACGACTTCCTCTCCGACCTCT	CGAGGCTCACGTAACCGTAGT
*Chrebpb*	TCTGCAGATCGCGTGGAG	CTTGTCCCGGCATAGCAAC
*Fkbp5*	ATTTGATTGCCGAGATGTG	TCTTCACCAGGGCTTTGTC
*Foxo1*	CTTCAAGGATAAGGGCGACA	GACAGATTGTGGCGAATTGA
*Irs1*	CGATGGCTTCTCAGACGTG	CAGCCCGCTTGTTGATGTTG
*Irs2*	GTGGGTTTCCAGAACGGCCT	ATGGGGCTGGTAGCGCTTCA
*Klf15*	GCGAGAAGCCCTTTGCCT	GCTTCACACCCGAGTGAGAT
*Lep*	ACCCCATTCTGAGTTTGTCC	TCCAGGTCATTGGCTATCTG
*Nr3c1*	CCGGGTCCCCAGGTAAAGA	TGTCCGGTAAAATAAGAGGCTTG
*Nr3c2*	ATGGAAACCACACGGTGACCT	AGCCTCATCTCCACACACCAAG
*Pck1*	ATGTGTGGGCGATGACATT	AACCCGTTTTCTGGGTTGAT
*Pcx*	GGGATGCCCACCAGTCACT	CATAGGGCGCAATCTTTTTGA
*Pparg*	GAAAGACAACGGACAAATCACC	GGGGGTGATATGTTTGAACTTG
*Slc2a1*	GACCCTGCACCTCATTGG	GATGCTCAGATAGGACATCCAAG
*Slc2a2*	CCAGTACATTGCGGACTTCCTT	CTTTCCTTTGGTTTCTGGAACTTT
*Slc2a4*	GTGACTGGAACACTGGTCCTA	CCAGCCACGTTGCATTGTAG
*Tsc22d3*	CAGCAGCCACTCAAACCAGC	ACCACATCCCCTCCAAGCAG
*Ucp1*	GGCCTCTACGACTCAGTCCA	TAAGCCGGCTGAGATCTTGT
*Actb*	AAGGCCAACCGTGAAAAGAT	GTGGTACGACCAGAGGCATAC
*B2m*	ATCCAAATGCTGAAGAACGG	CAGTCTCAGTGGGGGTGAAT
*Gapdh*	TGTCCGTCGTGGATCTGAC	CCTGCTTCACCACCTTCTTG
*Rn18s*	GTAACCCGTTGAACCCCATT	CCATCCAATCGGTAGTAGCG

### Protein extraction

Protein was extracted from the insulin-stimulated WAT explants by mincing the tissues with a micropestle in lysis buffer containing 50 mM HEPES, 150 mM NaCl, 10 mM EDTA, 2 mM Na_3_VO_4_, 20 mM NaF, 1% Triton X-100, phosphatase inhibitor (P5726, Sigma-Aldrich), and protease inhibitor (cOmplete, Roche Diagnostics). After centrifugation to remove debris, lysates were collected. For the cultured adipocytes, cells were lysed in the aforementioned lysis buffer and sonicated for 10 seconds. Protein concentrations were quantified relatively to BSA (Sigma-Aldrich) using the Advanced protein assay reagent (Cytoskeleton).

### Western blot analysis

Protein extracts were diluted in Laemmli sample buffer (Bio-Rad Laboratories, Veenendaal, Netherlands) with 50 mM dithiothreitol and denatured at 95°C for 5 minutes. A total of 15 µg of protein was electrophoresed on an 8% acrylamide gel and blotted onto a nitrocellulose membrane. Membranes were blocked in 3% skim milk powder in PBS for 1 hour at RT and incubated overnight at 4°C with an Akt antibody [1:1000 (35)] or a phosphorylated Akt (Ser473) antibody [1:2000 (36)] in PBS containing 0.1% Tween 20 and 5% BSA. Next, membranes were washed and incubated for 1 hour at RT with an IRDye 800CW goat anti-rabbit secondary antibody [1:10,000 (37)] in PBS containing 0.1% Tween 20 and 3% skim milk powder. The Akt and phosphorylated Akt immunoreactivities were detected with an Odyssey IR imaging system (LI-COR Biotechnology, Bad Homburg, Germany) and were quantified using Image Studio Lite software (version 5.2, LI-COR).

### Statistical analysis

Data were analyzed and graphs were plotted in GraphPad Prism for Windows (version 6, GraphPad Software, San Diego, CA) and IBM SPSS Statistics for Windows (version 24, IBM Corp., Armonk, NY). Unless otherwise indicated, differences between groups were analyzed by two-way ANOVA with a Tukey *post hoc* test when the interaction of factors was significant or with a Bonferroni test when the interaction was not significant. *P* < 0.05 was considered statistically significant [abbreviations for *P* values: *P*_C_ for corticosterone treatment, *P*_I_ for insulin treatment, *P*_S_ for sex, and *P*_T_ for time (duration) of treatment]. When analyzing the IPGTT data, the area under the curve (AUC) was calculated normalized to the fasting level of each animal (26, 38). Unless specified, data and graphs are shown as mean ± SEM.

## Results

### Corticosterone increases nonfasting glucose concentrations only in male mice

**Figure 2.**
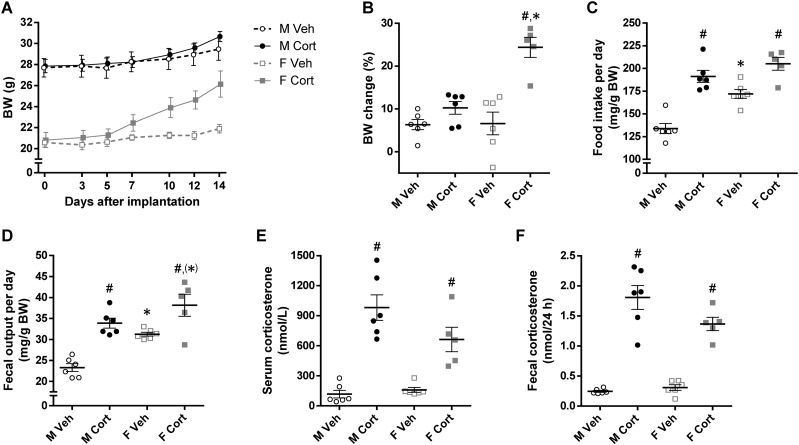
Effects of corticosterone treatment on BW and energy balance of mice. (A) BW of *ad libitum-*fed mice before (day 0) and after (days 3 to 14) pellet implantation (repeated three-way ANOVA: *P*_S_ < 0.001, *P*_C_ = 0.11, *P*_T_ < 0.001, *P*_S×C_ = 0.29, *P*_S×T_ = 0.02, *P*_C×T_ < 0.001, *P*_S×C×T_ < 0.001). (B) BW changes after 2-wk treatment relative to BW before pellet implantation (*P*_S_ = 0.002, *P*_C_ < 0.001, *P*_S×C_ = 0.002). (C) Daily food intake and (D) fecal production relative to BW, determined during days 12 and 14 of treatment (food intake, *P*_S_ < 0.001, *P*_C_ < 0.001, *P*_S×C_ = 0.06; fecal output, *P*_S_ < 0.001, *P*_C_ < 0.001, *P*_S×C_ = 0.20). (E) Serum corticosterone levels (level at time of euthanization: *P*_S_ = 0.14, *P*_C_ < 0.001, *P*_S×C_ = 0.06). (F) Fecal corticosterone levels (average level during days 12 to 14: *P*_S_ = 0.13, *P*_C_ < 0.001, *P*_S×C_ = 0.05). Unless stated, statistical significance was determined by two-way ANOVA. *P < 0.05, ^(^*^)^*P* < 0.10 (tendency to significance), for sex difference between mice with the same treatment; ^#^*P* < 0.05, for effect of corticosterone treatment in mice of the same sex, by *post hoc* test.

Corticosterone treatment for 2 weeks differentially affected body weight (BW) of male and female mice (experiment 1). The corticosterone-treated male mice were only 1.2 g heavier than vehicle-treated male mice whereas the corticosterone-treated female mice were 3.5 g heavier than vehicle-treated female mice (repeated three-way ANOVA: *P*_S×C×T_ < 0.001; Fig. 2A). Relative to BW at implantation, the corticosterone-treated female mice gained more weight than did the other three groups (*P*_S×C_ = 0.002; Fig. 2B). Although vehicle-treated female mice consumed more food (relative to BW) than did vehicle-treated male mice, corticosterone treatment significantly increased the 24-hour food intake to a comparable amount in both sexes (*P*_S_ < 0.001, *P*_C_ < 0.001; Fig. 2C). The 24-hour fecal output was higher in female than in male mice, was increased by corticosterone treatment (*P*_S_ < 0.001, *P*_C_ < 0.001; Fig. 2D), and was positively correlated with food intake (*r* = 0.93, *P* < 0.001; data not shown). Serum corticosterone levels (at time of euthanization) and fecal corticosterone levels (2-day average levels between days 12 and 14 of treatment) were measured and confirmed that corticosterone-treated mice had significantly higher corticosterone levels in serum and feces than did vehicle-treated mice (*P*_C_ < 0.001; Fig. 2E and 2F). Moreover, the corticosterone-treated male mice tended to have higher corticosterone levels than did corticosterone-treated female mice, but levels in vehicle-treated mice were not different between males and females (Fig. 2E and 2F).

**Figure 3.**
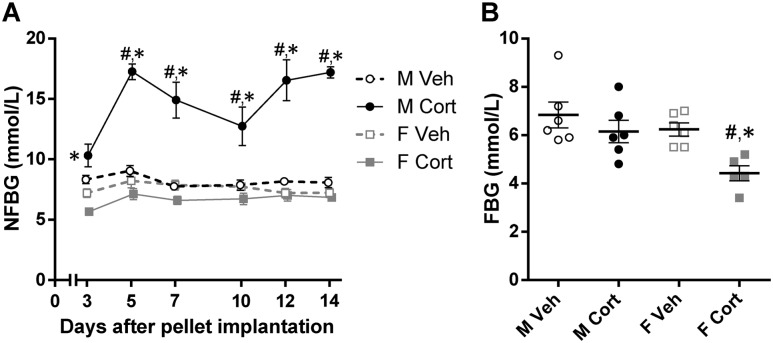
Sex-dependent effects of corticosterone treatment on glucose homeostasis. (A) NFBG levels during days 3 to 14 of treatment (repeated three-way ANOVA: *P*_S_ < 0.001, *P*_C_ < 0.001, *P*_T_ < 0.001, *P*_S×C_ < 0.001, *P*_S×T_ = 0.02, *P*_C×T_ < 0.001, *P*_S×C×T_ = 0.02; two-way ANOVA at each time point: *P*_S_ < 0.05 every time point, *P*_C_ = 0.69 for day 3, *P*_C_ < 0.05 for days 5 to 14, *P*_S×C_ < 0.05 every time point). (B) Fasting blood glucose levels determined on day 14 of treatment (*P*_S_ = 0.01, *P*_C_ = 0.008, *P*_S×C_ = 0.19). Unless stated, statistical significance was determined by two-way ANOVA. *P < 0.05, for sex difference between mice with the same treatment; ^#^*P* < 0.05, for effect of corticosterone treatment in mice of the same sex, by *post hoc* test.

Corticosterone treatment differentially affected NFBG of male and female mice. Corticosterone increased NFBG of male mice by approximately twofold whereas it had no effect on NFBG of female mice (repeated three-way ANOVA: *P*_S×C×T_ = 0.02; Fig. 3A). Measurement of 5-hour FBG after the 2-week treatment period revealed that female mice had a lower FBG than did male mice, and corticosterone treatment reduced the FBG in both sexes, but more pronounced in female mice (*P*_S_ = 0.01, *P*_C_ = 0.008; Fig. 3B). These data suggest that corticosterone treatment disturbs glucose homeostasis more severely in male than in female mice because only corticosterone-treated male mice presented with an elevated NFBG.

### Differential effects of corticosterone on glucose tolerance in male and female mice

**Figure 4.**
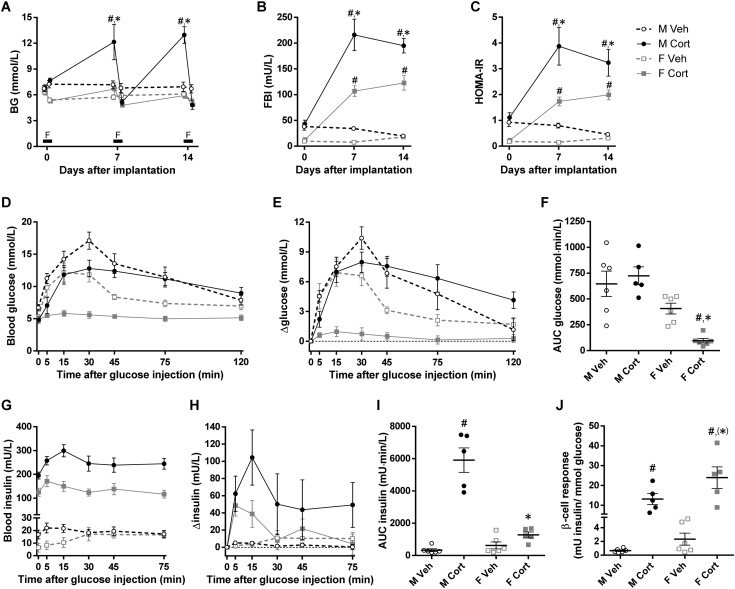
Sex-dependent effects of corticosterone treatment on glucose clearance. (A) Nonfasting and 5-h FBG levels (NFBG day 7, *P*_S_ = 0.003, *P*_C_ = 0.008, *P*_S×C_ = 0.06; FBG day 7, *P*_S_ = 0.22, *P*_C_ = 0.01, *P*_S×C_ = 0.60; NFBG day 14, *P*_S_ < 0.001, *P*_C_ < 0.001, *P*_S×C_ < 0.001; FBG day 14, *P*_S_ = 0.08, *P*_C_ = 0.009, *P*_S×C_ = 0.07). F indicates the 5-h fasting period on days 0, 7, and 14. (B) Five-hour FBI levels and (C) HOMA-IR calculated from FBG and insulin levels before (day 0) and after (days 7 and 14) pellet implantation (FBI day 7, *P*_S_ < 0.001, *P*_C_ < 0.001, *P*_S×C_ = 0.009; FBI day 14, *P*_S_ = 0.001, *P*_C_ < 0.001, *P*_S×C_ = 0.002; HOMA-IR day 7, *P*_S_ < 0.001, *P*_C_ < 0.001, *P*_S×C_ = 0.04; HOMA-IR day 14, *P*_S_ = 0.01, *P*_C_ < 0.001, *P*_S×C_ = 0.04). (D) Blood glucose levels, (E) changes in blood glucose levels over individual baseline values, (F) baseline-corrected AUCs of glucose levels, (G) blood insulin levels, (H) changes in blood insulin levels over individual baseline values, and (I) baseline-corrected AUCs of insulin levels after IP glucose administration in the 2-wk vehicle- or corticosterone-treated mice are shown (AUC glucose levels, *P*_S_ < 0.001, *P*_C_ = 0.16, *P*_S×C_ = 0.03; AUC insulin levels, *P*_S_ < 0.001, *P*_C_ < 0.001, *P*_S×C_ < 0.001). (J) *β*-Cell response to the IPGTT (*P*_S_ = 0.04, *P*_C_ < 0.001, *P*_S×C_ = 0.12). Statistical significance was determined by two-way ANOVA. *P < 0.05, ^(^*^)^*P* < 0.10 (tendency to significance), for sex difference between mice with the same treatment; ^#^*P* < 0.05, for effect of corticosterone treatment in mice of the same sex, by *post hoc* test.

To investigate the differential effects of high-dose corticosterone on blood glucose homeostasis in male and female mice in more detail, mice were subjected to an IPGTT on day 14 in the second experiment. Also in this experiment, corticosterone treatment differentially affected BW of male and female mice (data not shown) and increased NFBG only in male mice (NFBG day 14: *P*_S×C_ < 0.001; Fig. 4A). Compared with the vehicle-treated mice of the same sex, corticosterone treatment reduced the 5-hour FBG by 28.1% ± 7.4% in male mice and by 7.6% ± 4.6% in female mice (unpaired *t* test: *P* = 0.04; Fig. 4A). FBI was elevated in both sexes after 7 and 14 days of corticosterone treatment, and the effect was more pronounced in male than in female mice (*P*_S×C_ < 0.01 for both days; Fig. 4B). Likewise, corticosterone treatment increased the homeostatic model assessment of insulin resistance (HOMA-IR) values more profoundly in male mice than in female mice (*P*_S×C_ < 0.05 for both days; Fig. 4C). These data underscore that corticosterone-induced insulin resistance is more pronounced in male than in female mice.

After the IP administration of glucose, peak glucose levels of corticosterone-treated male mice were lower than in vehicle-treated males. However, corticosterone-treated male mice showed a delayed glucose clearance, and glucose levels did not return to baseline levels (Fig. 4D and 4E). As a result, baseline-corrected AUC values were slightly but not significantly increased in the corticosterone-treated males (*P*_S×C_ = 0.03; Fig. 4F). In contrast, in corticosterone-treated female mice the blood glucose levels were blunted, resulting in a significantly reduced baseline-corrected AUC value (Fig. 4D–4F). Blood insulin levels after IPGTT were markedly different between the groups, mainly due to the elevated baseline FBI upon corticosterone treatment (Fig. 4G). Interestingly, the rise in blood insulin levels upon glucose injection was greater in the corticosterone-treated male mice than in the other three groups (*P*_S×C_ < 0.001; Fig. 4H and 4I). Calculated from the glucose and insulin AUC, the *β*-cell response was largely increased by corticosterone treatment and was higher in female mice than in male mice (*P*_S_ = 0.04, *P*_C_ < 0.001; Fig. 4J).

**Figure 5.**
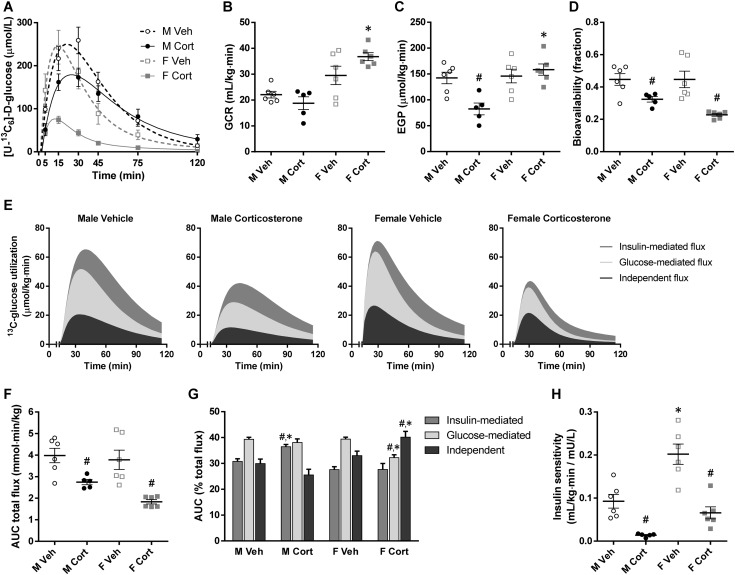
Glucose kinetic parameters from stable isotope-labeled glucose analyses. (A) Blood [U-^13^C_6_]-d-glucose levels with model-fitted line plots after IP stable isotope-labeled glucose administration in the 2-wk vehicle- or corticosterone-treated mice. This plot was used for calculating following glucose kinetic parameters. (B) Glucose clearance rate and (C) endogenous glucose production at basal state (before glucose administration; GCR, *P*_S_ < 0.001, *P*_C_ = 0.42, *P*_S×C_ = 0.04; EGP, *P*_S_ = 0.003, *P*_C_ = 0.06, *P*_S×C_ = 0.006). (D) Bioavailability of the injected glucose in the accessible pool of the minimal mouse model (*P*_S_ = 0.18, *P*_C_ < 0.001, *P*_S×C_ = 0.18). (E) Stacked area plots demonstrating utilization of the injected glucose, separated into independent, glucose-mediated, and insulin-mediated fluxes. (F) AUCs of total glucose utilization (AUC total flux, *P*_S_ = 0.08, *P*_C_ < 0.001, *P*_S×C_ = 0.26). (G) Relative percentages of each glucose utilization flux (*P*_S×C_ < 0.05 for all fluxes). (H) Insulin sensitivity reflecting the clearance of injected glucose by endogenous insulin secretion (*P*_S_ < 0.001, *P*_C_ < 0.001, *P*_S×C_ = 0.10). Statistical significance was determined by two-way ANOVA. *P < 0.05, for sex difference between mice with the same treatment; ^#^*P* < 0.05, for effect of corticosterone treatment in mice of the same sex, by *post hoc* test.

Using the enrichment of blood [U-^13^C_6_]-d-glucose (Fig. 5A), the effects of corticosterone treatment on the glucose clearance rate (GCR) and the EGP at baseline (before glucose injection) can be calculated. The GCR was higher in female mice than in male mice, and corticosterone treatment tended to sex-dependently affect GCR, namely a reduction in male mice and an increase in female mice (*P*_S×C_ = 0.04; Fig. 5B). The EGP was reduced by corticosterone treatment in male mice but was unaffected in female mice (*P*_S×C_ = 0.006; Fig. 5C). The factors contributing to blood glucose levels after the injected bolus can also be assessed. First, the bioavailability of the injected glucose was reduced by corticosterone treatment, but sex-independently (*P*_C_ < 0.001; Fig. 5D). Second, detailed analysis of the clearance of the injected glucose revealed that the glucose-mediated flux, the insulin-mediated flux, and the independent flux were in general reduced by corticosterone treatment in both male and female mice (AUC total flux: *P*_C_ < 0.001; Fig. 5E and 5F). Interestingly, the percentage contribution of each flux was sex-dependently altered by corticosterone treatment, shifting toward insulin-mediated flux for male mice but toward independent flux for female mice (*P*_S×C_ < 0.05 for all fluxes; Fig. 5G). Finally, the insulin sensitivity index (insulin-mediated glucose clearance) was markedly reduced by corticosterone treatment, and female mice had a greater insulin sensitivity index than did male mice (*P*_S_ < 0.001, *P*_C_ < 0.001; Fig. 5H). This finding once again underscores that corticosterone-induced insulin resistance is more pronounced in male mice than in female mice.

### Sex-differential effects of corticosterone on WAT morphology

**Figure 6.**
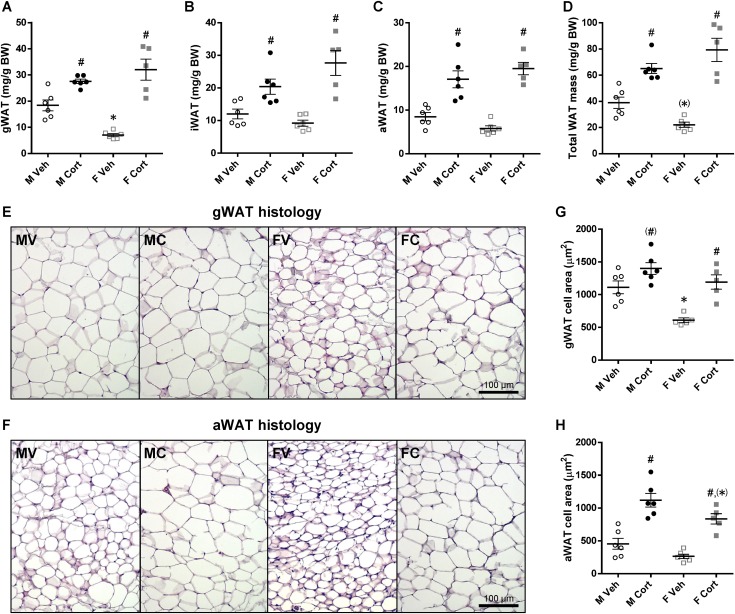
Sex differences in WAT mass and morphology upon corticosterone treatment. (A–D) gWAT, iWAT, aWAT, and total WAT mass relative to BW of the 2-wk vehicle- or corticosterone-treated mice (gWAT, *P*_S_ = 0.12, *P*_C_ < 0.001, *P*_S×C_ = 0.001; iWAT, *P*_S_ = 0.33, *P*_C_ < 0.001, *P*_S×C_ = 0.04; aWAT, *P*_S_ = 0.93, *P*_C_ < 0.001, *P*_S×C_ = 0.07; total WAT mass, *P*_S_ = 0.80, *P*_C_ < 0.001, *P*_S×C_ = 0.006). (E and F) Hematoxylin and eosin–stained gWAT and aWAT. Experimental group abbreviations: FC, female corticosterone; FV, female vehicle; MC, male corticosterone; MV, male vehicle. Scale bars, 100 µm. (G and H) gWAT and aWAT adipocyte sizes (gWAT, *P*_S_ < 0.001, *P*_C_ < 0.001, *P*_S×C_ = 0.12; aWAT, *P*_S_ = 0.008, *P*_C_ < 0.001, *P*_S×C_ = 0.58). Statistical significance was determined by two-way ANOVA. *P < 0.05, ^(^*^)^*P* < 0.10 (tendency to significance), for sex difference between mice with the same treatment; ^#^*P* < 0.05, for effect of corticosterone treatment in mice of the same sex, by *post hoc* test.

Adipose tissue is one of the glucose-consuming tissues, and we previously reported marked effects of corticosterone treatment on adipose tissue depots in male mice (39). Because the current data suggest that glucose clearance is differentially affected by corticosterone treatment in male and female mice, we next investigated the effects of corticosterone on male and female adipose tissues in more detail. Corticosterone treatment altered many aspects of WAT and BAT morphology and function with some clear differences between male and female mice. The visceral depot gWAT had a substantially greater mass in vehicle-treated male mice than in vehicle-treated female mice. This sex-dependent pattern disappeared after corticosterone treatment, as corticosterone-treated male and female mice had a comparable gWAT mass (*P*_S×C_ = 0.001; Fig. 6A). Two subcutaneous depots, iWAT and aWAT, also gained more mass upon corticosterone treatment, but there was no significant sex difference (*P*_C_ < 0.001 for both depots, *P*_S×C_ = 0.04 for iWAT; Fig. 6B and 6C). Corticosterone treatment noticeably elevated the total WAT mass (the sum of the aforementioned WAT masses) without a significant sex difference (*P*_S×C_ = 0.006; Fig. 6D).

Because the mode of WAT expansion (hypertrophy or hyperplasia) can explain WAT functions and adaptations, the histologies of gWAT (Fig. 6E) and aWAT (Fig. 6F) after corticosterone treatment were studied. Female mice had a remarkably smaller gWAT adipocyte size than did male mice, and corticosterone treatment enlarged gWAT adipocyte size in both sexes (*P*_S_ < 0.001, *P*_C_ < 0.001; Fig. 6G). The cross-sectional area of aWAT adipocytes was also smaller in female mice than in male mice, and corticosterone treatment increased the aWAT adipocyte size in both sexes (*P*_S_ = 0.008, *P*_C_ < 0.001; Fig. 6H).

Corticosterone treatment induces significant changes in gene expression in a partly depot- and sex-dependent manner. The mRNA expression of *Nr3c1* encoding the GC receptor (GR) in gWAT was lower in female mice than in male mice, whereas its expression in iWAT was higher in female mice than in male mice. Corticosterone treatment reduced *Nr3c1* mRNA expression in both depots of both sexes (Table 2). The mRNA expression of the GR target genes *Fkbp5* and *Tsc22d3* was significantly induced by corticosterone treatment in both depots with a general trend of higher expression in corticosterone-treated male depots than in female depots (Table 2). Although corticosterone and sex of the mice did not affect the mRNA expression of *Nr3c2* encoding the mineralocorticoid receptor (MR) in gWAT, its expression in iWAT was strikingly higher in vehicle-treated female mice than in male mice. However, this sex-differential pattern in iWAT disappeared upon corticosterone treatment because it reduced *Nr3c2* mRNA expression in female mice only (Table 2).

**Table 2.** mRNA Expression in gWAT and iWAT

Genes	gWAT					iWAT				
	Sig.	M Veh	M Cort	F Veh	F Cort	Sig.	M Veh	M Cort	F Veh	F Cort
GR, MR, and GR target genes										
* Nr3c1*	S,C	1.00 ± 0.16	0.46 ± 0.04^*a*^	0.58 ± 0.08^*b*^	0.34 ± 0.04	S,C,×	1.00 ± 0.06	0.67 ± 0.04^*a*^	1.78 ± 0.13^*b*^	0.41 ± 0.05^*a*^^,^^*b*^
* Fkbp5*	S,C,×	1.00 ± 0.28	6.90 ± 0.81^*a*^	0.38 ± 0.02	3.57 ± 0.84^*a*^^,^^*b*^	S,C,×	1.00 ± 0.26	10.46 ± 1.49^*a*^	2.58 ± 0.83	3.53 ± 0.81^*b*^
* Tsc22d3*	C	1.00 ± 0.33	6.31 ± 0.34^*a*^	0.59 ± 0.11	5.82 ± 1.05^*a*^	C,×	1.00 ± 0.34	6.39 ± 0.57^*a*^	1.51 ± 0.32	4.53 ± 0.34^*a*^^,^^*b*^
* Nr3c2*	—	1.00 ± 0.32	0.51 ± 0.12	0.62 ± 0.08	0.72 ± 0.19	S,C,×	1.00 ± 0.06	0.80 ± 0.26	6.83 ± 2.76^*b*^	0.85 ± 0.25^*a*^
Adipogenic differentiation markers										
* Cebpb*	C	1.00 ± 0.12	3.78 ± 0.86^*a*^	0.90 ± 0.16	2.61 ± 0.43^*c*^	C	1.00 ± 0.18	1.76 ± 0.13^*a*^	1.41 ± 0.32	2.13 ± 0.28^*c*^
* Pparg*	(C)	1.00 ± 0.23	1.46 ± 0.16	1.10 ± 0.21	1.65 ± 0.36	(S)	1.00 ± 0.26	1.12 ± 0.08	1.51 ± 0.34	1.50 ± 0.23
* Adamts1*	S,C	1.00 ± 0.10	2.48 ± 0.23^*a*^	0.40 ± 0.07^*b*^	0.81 ± 0.11^*a*^^,^^*b*^	C,×	1.00 ± 0.26	7.17 ± 0.84^*a*^	3.06 ± 0.80^*d*^	4.02 ± 0.71^*b*^
Adipokine production										
* Lep*	S,C,×	1.00 ± 0.01	24.19 ± 4.55^*a*^	0.22 ± 0.01^*b*^	9.63 ± 1.24^*a*^^,^^*b*^	C,×	1.00 ± 0.37	9.45 ± 1.39^*a*^	1.97 ± 0.69	5.83 ± 0.83^*a*^
* Adipoq*	C	1.00 ± 0.16	0.68 ± 0.07	1.09 ± 0.15	0.58 ± 0.11^*a*^	(S),C	1.00 ± 0.20	0.57 ± 0.09	1.93 ± 0.52^*d*^	0.90 ± 0.26^*c*^
Glucose transport										
* Irs1*	—	1.00 ± 0.12	0.67 ± 0.14	1.24 ± 0.18	1.11 ± 0.46	C	1.00 ± 0.21	0.33 ± 0.10^*a*^	1.04 ± 0.35	0.42 ± 0.12^*c*^
* Slc2a1*	S,(C)	1.00 ± 0.31	1.30 ± 0.19	0.31 ± 0.06	0.88 ± 0.25	C	1.00 ± 0.41	0.29 ± 0.04^*a*^	0.97 ± 0.14	0.51 ± 0.12
* Slc2a4*	C	1.00 ± 0.23	3.21 ± 0.92^*c*^	0.88 ± 0.23	5.34 ± 1.22^*a*^	(C)	1.00 ± 0.18	1.55 ± 0.20	0.97 ± 0.39	1.30 ± 0.16
* Chrebpb*	S,C	1.00 ± 0.37	5.96 ± 2.12	5.41 ± 1.94^*b*^	15.82 ± 3.59^*a*^	C,×	1.00 ± 0.40	1.04 ± 0.28	0.18 ± 0.13	1.82 ± 0.36^*a*^

Gene expression was normalized to *B2m* and *Rn18s* expression and expressed relative to the vehicle-treated male mice. Sig. indicates significant effects (*P* < 0.05) analyzed with two-way ANOVA. Symbols in parentheses [*e.g.*, (C)] indicate a tendency to significance (*P* < 0.10).

Abbreviations: C, corticosterone treatment; S, sex; ×, interaction of corticosterone treatment and sex.

^a^ *P* < 0.05, for effect of corticosterone treatment in mice of the same sex, by *post hoc* test.

^b^ *P* < 0.05, for sex difference between mice with the same treatment, by *post hoc* test.

^c^ *P* < 0.10 (tendency to significance), for effect of corticosterone treatment in mice of the same sex, by *post hoc* test.

^d^ *P* < 0.10 (tendency to significance), for sex difference between mice with the same treatment, by *post hoc* test.

In gWAT, mRNA expression of the adipogenic transcription factors *Cebpb* and *Pparg* was generally elevated by corticosterone treatment in both sexes. In iWAT, expression of both transcription factors tended to be higher in female mice than in male mice (Table 2). ADAMTS1, an extracellular protein secreted from mature adipocytes, together with *Adamts1* mRNA expression has been considered a marker of the hyperplastic limitation in WAT because it inhibits the generation of new adipocytes from adipocyte precursor cells (40). In gWAT, female mice had a lower *Adamts1* mRNA expression than did male mice, and corticosterone treatment induced its expression in both sexes. However, corticosterone treatment induced *Adamts1* mRNA expression by sevenfold in iWAT of male mice but did not significantly affect the expression in female mice (Table 2). Of note, *Adamts1* mRNA expression in gWAT was on average 11-fold higher than in iWAT (data not shown).

### Sex-differential effects of corticosterone on adipokine secretion

**Figure 7.**
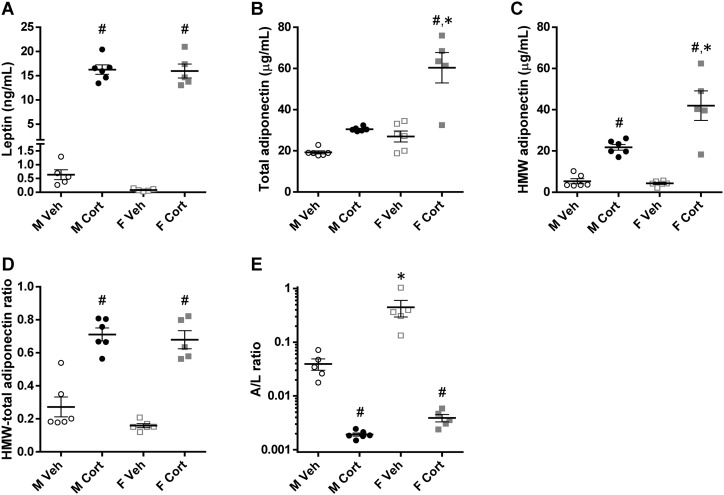
Serum adipokine levels. (A) Serum leptin levels and (B and C) serum total adiponectin and HMW isoform levels of the 2-wk vehicle- or corticosterone-treated mice (leptin, *P*_S_ = 0.64, *P*_C_ < 0.001, *P*_S×C_ = 0.89; total adiponectin, *P*_S_ < 0.001, *P*_C_ < 0.001, *P*_S×C_ = 0.005; HMW adiponectin, *P*_S_ = 0.008, *P*_C_ < 0.001, *P*_S×C_ = 0.004). (D) Ratio of the HMW isoform to the total adiponectin levels (*P*_S_ = 0.12, *P*_C_ < 0.001, *P*_S×C_ = 0.37). (E) Ratio of adiponectin to leptin levels, illustrated on a logarithmic scale (*P*_S_ < 0.001, *P*_C_ < 0.001, *P*_S×C_ = 0.007). Statistical significance was determined by two-way ANOVA. *P < 0.05, for sex difference between mice with the same treatment; ^#^*P* < 0.05, for effect of corticosterone treatment in mice of the same sex, by *post hoc* test.

Apart from energy storage as lipid droplets, adipose tissue depots secrete a number of adipokines into the circulation. Serum leptin concentrations were strongly elevated after corticosterone treatment in both male and female mice (*P*_C_ < 0.001; Fig. 7A). The serum concentrations of total adiponectin were in general higher in female mice than in male mice and were increased upon corticosterone treatment, especially in female mice (*P*_S×C_ = 0.005; Fig. 7B). Serum concentrations of HMW adiponectin, the metabolically active adiponectin isoform, were also elevated by corticosterone treatment (*P*_S×C_ = 0.004; Fig. 7C). The HMW/total adiponectin ratio did not differ between the sexes but was significantly higher after corticosterone treatment (*P*_C_ < 0.001; Fig. 7D). The adiponectin/leptin (A/L) ratio, a promising index for assessing insulin sensitivity (41), was higher in vehicle-treated female mice than in male mice, but this sex difference was attenuated and reduced to a similar low A/L ratio after corticosterone treatment (*P*_S×C_ = 0.007; Fig. 7E). The HMW adiponectin/leptin ratio showed a similar pattern (*P*_S_ < 0.001, *P*_C_ < 0.001, *P*_S×C_ = 0.001; data not shown).

The changes in leptin concentrations were also reflected by changes in gene expression. Corticosterone treatment induced *Lep* mRNA expression in gWAT and iWAT of both sexes, which was more pronounced in corticosterone-treated male mice than in female mice (Table 2). In contrast, corticosterone treatment reduced *Adipoq* mRNA expression in both depots of both sexes. Furthermore, female mice tended to have a higher *Adipoq* mRNA expression in iWAT than did male mice (Table 2). Note that *Lep* mRNA expression in gWAT was on average fivefold higher whereas *Adipoq* mRNA expression was 0.7-fold lower than in iWAT (data not shown).

### Corticosterone treatment reduces BAT activity in both male and female mice

**Figure 8.**
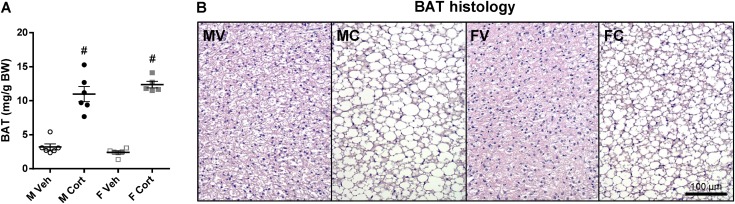
BAT mass and morphology upon corticosterone treatment. (A) BAT mass relative to BW of the 2-wk vehicle- or corticosterone-treated mice (*P*_S_ = 0.68, *P*_C_ < 0.001, *P*_S×C_ = 0.12). (B) Hematoxylin and eosin–stained BAT. Experimental group abbreviations: FC, female corticosterone; FV, female vehicle; MC, male corticosterone; MV, male vehicle. Scale bar, 100 µm. Statistical significance was determined by two-way ANOVA. ^#^*P* < 0.05, for effect of corticosterone treatment in mice of the same sex, by *post hoc* test.

In both male and female mice, corticosterone treatment increased BAT mass (*P*_C_ < 0.001; Fig. 8A) and induced lipid accumulation and unilocular rearrangement in BAT (Fig. 8B). The mRNA expression of *Ucp1*, a classical BAT thermogenic gene, was reduced by corticosterone treatment in both sexes (Table 3). Although corticosterone treatment tended to reduce mRNA expression of *Nr3c1* without a sex-dependent pattern in BAT, it significantly induced expression of the GR target genes *Fkbp5* and *Tsc22d3* in both sexes, albeit less pronounced in female mice than in male mice (Table 3). The mRNA expression of *Nr3c2* was lower in female mice than in male mice without an obvious effect by corticosterone treatment (Table 3).

**Table 3.** mRNA Expression in BAT

Genes	Sig.	M Veh	M Cort	F Veh	F Cort
Thermogenic gene					
* Ucp1*	C,×	1.00 ± 0.16	0.80 ± 0.21	1.61 ± 0.25	0.50 ± 0.15^*a*^
GR, MR, and GR target genes					
* Nr3c1*	(C)	1.00 ± 0.11	0.86 ± 0.10	0.97 ± 0.13	0.68 ± 0.09
* Fkbp5*	S,C,×	1.00 ± 0.12	43.82 ± 4.34^*a*^	0.64 ± 0.06	30.00 ± 4.79^*a*^^,^^*b*^
* Tsc22d3*	C,×	1.00 ± 0.08	5.05 ± 0.35^*a*^	1.36 ± 0.13	3.89 ± 0.41^*a*^^,^^*b*^
* Nr3c2*	S	1.00 ± 0.07	0.89 ± 0.11	0.59 ± 0.12^*b*^	0.76 ± 0.15
Adipokine production					
* Lep*	(S),C	1.00 ± 0.36	38.56 ± 7.49^*a*^	0.12 ± 0.03	23.54 ± 4.55^*a*^^,^^*c*^
* Adipoq*	—	1.00 ± 0.10	0.98 ± 0.14	1.23 ± 0.15	0.82 ± 0.09
Glucose transport					
* Irs1*	—	1.00 ± 0.21	1.19 ± 0.11	2.10 ± 0.78	1.12 ± 0.21
* Slc2a1*	—	1.00 ± 0.15	1.23 ± 0.19	0.74 ± 0.09	1.00 ± 0.15
* Slc2a4*	—	1.00 ± 0.17	1.00 ± 0.13	0.84 ± 0.08	1.10 ± 0.10
* Chrebpb*	×	1.00 ± 0.19	0.63 ± 0.13	0.44 ± 0.07^*c*^	0.92 ± 0.18

Gene expression was normalized to *Actb* and *Rn18s* expression and expressed relative to the vehicle-treated male mice. Sig. indicates significant effects (*P* < 0.05) analyzed with two-way ANOVA. Symbols in parentheses [*e.g.*, (C)] indicate a tendency to significance (*P* < 0.10).

Abbreviations: C, corticosterone treatment; S, sex; ×, interaction of corticosterone treatment and sex.

^a^ *P* < 0.05, for effect of corticosterone treatment in mice of the same sex, by *post hoc* test.

^b^ *P* < 0.05, for sex difference between mice with the same treatment, by *post hoc* test.

^c^ *P* < 0.10 (tendency to significance), for sex difference between mice with the same treatment, by *post hoc* test.

*Lep* mRNA expression in BAT of female mice tended to be lower than that of male mice, and corticosterone treatment strongly induced the expression in both sexes by >35-fold (Table 3). However, *Adipoq* mRNA expression in BAT was not significantly affected by corticosterone treatment in both sexes (Table 3).

### Elevated concentrations of leptin and adiponectin are likely due to hyperinsulinemia, not a direct effect of corticosterone

To investigate whether the elevated serum leptin and adiponectin levels upon corticosterone treatment are caused directly by corticosterone or indirectly as a result of the compensatory hyperinsulinemia, *in vitro* studies using 3T3-L1 white adipocytes and T37i brown adipocytes were performed. In 3T3-L1 cells, corticosterone reduced whereas insulin induced *Lep* mRNA expression (Table 4). Leptin production and secretion by 3T3-L1 cells were also significantly stimulated by insulin treatment (Table 4). Although both insulin and corticosterone reduced *Adipoq* mRNA expression in 3T3-L1 cells, cotreatment of insulin and corticosterone attenuated the inhibitory effect of corticosterone on *Adipoq* mRNA expression (Table 4). In contrast, insulin significantly increased whereas corticosterone reduced the total adiponectin production in 3T3-L1 cells, and adiponectin secretion tended to be increased by corticosterone only (Table 4).

**Table 4.** Leptin and Adiponectin Gene Expression and Adipokine Production in Cultured Adipocytes

Adipokine	3T3-L1 cells					T37i cells				
	Sig.	Control	CORT	INS	CORT+INS	Sig.	Control	CORT	INS	CORT+INS
*Lep* mRNA expression	C,I,×	1.00 ± 0.32	0.26 ± 0.06^*a*^	1.61 ± 0.31	1.59 ± 0.25^*b*^	C,I,×	1.00 ± 0.14	0.30 ± 0.04^*a*^	8.54 ± 2.39^*b*^	8.27 ± 1.27^*b*^
Leptin, pg/g protein										
In cell lysates	(I)	40 ± 7	43 ± 9	59 ± 4	52 ± 4	I	285 ± 52	259 ± 59	181 ± 20	178 ± 23
In cultured media	I	117 ± 12	98 ± 15	575 ± 127^*b*^	896 ± 237^*b*^	C,I	379 ± 34	204 ± 23^*a*^	2851 ± 539^*b*^	2368 ± 320^*b*^
Total production	I	157 ± 11	141 ± 10	634 ± 126^*b*^	949 ± 237^*b*^	(C),I	664 ± 43	463 ± 55^*c*^	3032 ± 523^*b*^	2546 ± 304^*b*^
Leptin secretion, %	I	74.0 ± 4.1	68.1 ± 8.1	89.5 ± 1.9^*d*^	91.6 ± 2.4^*b*^	I	58.2 ± 6.4	47.2 ± 7.2	92.7 ± 1.7^*b*^	91.9 ± 2.1^*b*^
*Adipoq* mRNA expression	C,×	1.00 ± 0.08	0.34 ± 0.03^*a*^	0.69 ± 0.06^*b*^	0.62 ± 0.03^*b*^	C,I,×	1.00 ± 0.06	0.13 ± 0.02^*a*^	0.80 ± 0.06	0.43 ± 0.04^*a*^^,^^*b*^
Adiponectin, ng/g protein										
In cell lysates	C,I	2285 ± 112	1810 ± 219^*c*^	2990 ± 125^*b*^	2524 ± 137^*b*^^,^^*c*^	C,I	750 ± 41	315 ± 20^*a*^	1139 ± 94^*b*^	733 ± 75^*a*^^,^^*b*^
In cultured media	I	1437 ± 103	1493 ± 140	1801 ± 164	1667 ± 64	C,I	1061 ± 91	775 ± 88^*a*^	766 ± 31^*b*^	520 ± 29^*a*^^,^^*b*^
Total production	C,I	3723 ± 104	3303 ± 262	4791 ± 261^*b*^	4192 ± 144^*b*^	C	1811 ± 127	1090 ± 87^*a*^	1905 ± 78	1253 ± 89^*a*^
Adiponectin secretion, %	(C)	38.6 ± 2.5	46.3 ± 3.7^*c*^	37.1 ± 1.7	40.0 ± 1.8	C,I,×	58.3 ± 1.3	70.2 ± 2.7^*a*^	40.6 ± 2.8^*b*^	42.1 ± 2.4^*b*^

Gene expression was normalized to *Actb* and *B2m* expression and expressed relative to the control condition. Sig. indicates significant effects (*P* < 0.05) analyzed with two-way ANOVA. Symbols in parentheses [*e.g.*, (I)] indicate a tendency to significance (*P* < 0.10).

Abbreviations: C, corticosterone; CORT, corticosterone treatment; CORT+INS, cotreatment with corticosterone and insulin; I, insulin; INS, insulin treatment; ×, interaction of corticosterone and insulin.

^a^ *P* < 0.05, for effect of corticosterone within the same insulin treatment, by *post hoc* test.

^b^ *P* < 0.05, for effect of insulin within the same corticosterone treatment, by *post hoc* test.

^c^ *P* < 0.10 (tendency to significance), for effect of corticosterone within the same insulin treatment, by *post hoc* test.

^d^ *P* < 0.10 (tendency to significance), for effect of insulin within the same corticosterone treatment, by *post hoc* test.

In T37i cells, corticosterone reduced whereas insulin strongly induced *Lep* mRNA expression (Table 4). Likewise, leptin production and secretion by T37i cells were stimulated by insulin treatment and cotreatment of corticosterone and insulin, while corticosterone treatment alone marginally reduced its production (Table 4). Although corticosterone reduced *Adipoq* and insulin did not alter *Adipoq* mRNA expression in T37i cells, cotreatment of insulin and corticosterone attenuated the inhibitory effect of corticosterone treatment (Table 4). Furthermore, whereas insulin treatment only increased intracellular adiponectin but did not affect the total production, corticosterone treatment reduced total adiponectin production but increased its secretion significantly (Table 4). Apart from adiponectin production in brown adipocytes, these data suggest that the elevated adipokine levels of corticosterone-treated mice were more likely caused by the high insulin level than by corticosterone treatment itself.

### Corticosterone treatment reduces insulin-stimulated Akt phosphorylation in WAT

**Figure 9.**
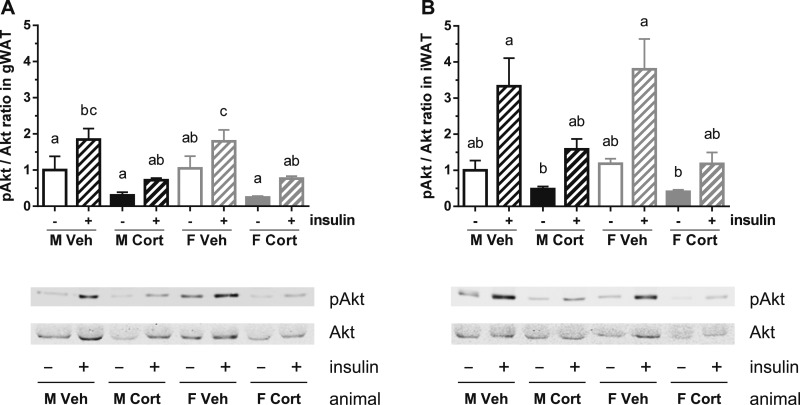
Insulin-stimulated Akt phosphorylation in WAT explants. (A and B) Akt phosphorylation level in (A) gWAT and (B) iWAT of the vehicle- or corticosterone-treated mice, *ex vivo* stimulated with insulin (gWAT, *P*_S_ = 0.99, *P*_C_ = 0.005, *P*_I_ < 0.001, *P*_S×C_ = 0.99, *P*_S×I_ = 0.95, *P*_C×I_ = 0.02, *P*_S×C×I_ = 0.39; iWAT, *P*_S_ = 0.91, *P*_C_ = 0.004, *P*_I_ < 0.001, *P*_S×C_ = 0.45, *P*_S×I_ = 0.97, *P*_C×I_ = 0.02, *P*_S×C×I_ = 0.57). Akt phosphorylation level was normalized to total Akt and expressed relative to the level of vehicle-treated male explants. A representative blot is shown of three biological samples per group. Statistical significance was determined by repeated three-way ANOVA with *post hoc* Tukey test: letters a, b, and c denote significant group differences (*P* < 0.05).

Insulin induces glucose uptake in WATs through Akt phosphorylation and subsequently GLUT4 translocation (42). To determine insulin signaling in WATs of vehicle- and corticosterone-treated mice, gWAT (a visceral depot) and iWAT (a subcutaneous depot) explants were stimulated with insulin and Akt phosphorylation was determined. Corticosterone treatment reduced baseline and insulin-stimulated Akt phosphorylation without a sex-dependent pattern in both gWAT and iWAT explants (repeated 3-way ANOVA: *P*_C×I_ < 0.05 in both explants; Fig. 9).

### Corticosterone reduces whereas insulin induces glucose uptake, but both cause insulin resistance in WAT

To address the effects of corticosterone treatment on adipose tissue glucose homeostasis, we assessed the adipose tissue mRNA expression of genes related to glucose transport. Corticosterone treatment did not affect mRNA expression of *Irs1*, the gene encoding the insulin receptor, in gWAT and BAT, but it reduced its expression in iWAT (Tables 2 and 3). In gWAT, the mRNA expression of the glucose transporter 4 [*Slc2a4* (*Glut4*)], an insulin-dependent glucose transporter, and the glucose transporter 1 [*Slc2a1* (*Glut1*)], a basal glucose transporter, were in general increased by corticosterone treatment (Table 2). In iWAT, corticosterone treatment reduced *Slc2a1* expression but tended to increase *Slc2a4* expression (Table 2). In contrast, corticosterone treatment did not significantly affect *Slc2a1* and *Slc2a4* mRNA expression in BAT (Table 3). Finally, we analyzed the expression of *Mlxipl* variant *β* (or called *Chrebpb*), a gene whose transcription is regulated by the carbohydrate-responsive element-binding protein (ChREBP; also known as MLX interacting protein-like) because it can be considered a readout of intracellular glucose concentrations (43). Corticosterone treatment increased *Chrebpb* expression in gWAT and iWAT, but not in BAT, suggestive of an increased glucose uptake after corticosterone treatment in both WAT depots (Tables 2 and 3). Of note, *Chrebpb* expression in the iWAT depot was significantly induced by corticosterone treatment only in female mice due to the sex-differential baseline expression.

**Figure 10.**
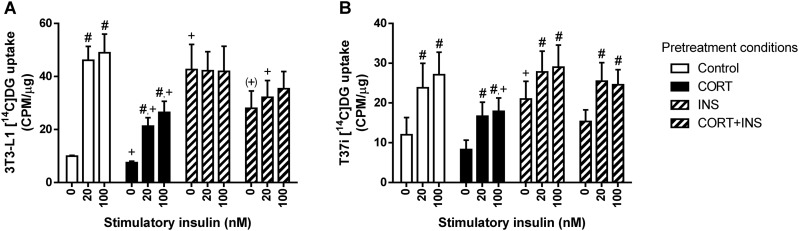
Insulin-stimulated radioactive glucose uptake in corticosterone- and/or insulin-treated 3T3-L1 and T37i adipocytes. (A) Differentiated 3T3-L1 white adipocytes and (B) differentiated T37i brown adipocytes pretreated with corticosterone (PC) and/or insulin (PI) for 24 h were stimulated with insulin (SI) at the indicated concentrations for 15 min, and subsequently 2-[1-^14^C]-deoxy-d-glucose was added to determine glucose uptake (3T3-L1, *P*_PC_ = 0.03, *P*_PI_ = 0.09, *P*_SI_ < 0.001, *P*_PC×PI_ = 0.60, *P*_PC×SI_ = 0.08, *P*_PI×SI_ < 0.001, *P*_PC×PI×SI_ = 0.001; T37i, *P*_PC_ = 0.22, *P*_PI_ = 0.16, *P*_SI_ < 0.001, *P*_C×PI_ = 0.77, *P*_PC×SI_ = 0.46, *P*_PI×SI_ = 0.17, *P*_PC×PI×SI_ = 0.15). Data from three independent experiment were plotted in counts per minute, relative to protein content (µg) of each sample. Pretreatment condition abbreviations: CORT, corticosterone; CORT+INS, cotreatment with corticosterone and insulin; INS, insulin. Statistical significance was determined by repeated three-way ANOVA. ^+^*P* < 0.05, ^(+)^*P* < 0.10 (tendency to significance), for difference from baseline uptake of control condition; ^#^*P* < 0.05, for effect of the stimulatory insulin from its baseline uptake, by *post hoc* Dunnett test.

To determine whether the increased uptake of glucose in WAT upon corticosterone treatment is due to corticosterone *per se* and/or the high plasma insulin concentrations, we measured the insulin-stimulated glucose uptake in 3T3-L1 and T37i cells pretreated for 24 hours with corticosterone and/or insulin. In 3T3-L1 cells, pretreatment with corticosterone (PC) inhibited whereas pretreatment with insulin (PI) stimulated basal glucose uptake (*P*_PC×PI_ = 0.009; Fig. 10A). Note, however, that the co-pretreatment of corticosterone and insulin resulted in a 34% lower basal glucose uptake than did insulin pretreatment alone. The insulin-stimulated glucose uptake pattern was affected by pretreatment with corticosterone, pretreatment with insulin, and stimulatory insulin (SI) (repeated three-way ANOVA: *P*_PC×PI×SI_ = 0.001; Fig. 10A). Pretreatment with corticosterone and insulin alone or in combination significantly inhibited the insulin-induced glucose uptake (Fig. 10A).

For T37i cells, the basal glucose uptake was decreased by pretreatment with corticosterone but increased by pretreatment with insulin (*P*_PC_ = 0.05, *P*_PI_ < 0.001; Fig. 10B). The insulin-stimulated glucose uptake pattern was increased by stimulatory insulin, but it was not significantly altered by pretreatment with corticosterone or pretreatment with insulin, reflecting an insulin-sensitive state of the corticosterone- or insulin-pretreated T37i cells (repeated three-way ANOVA: *P*_SI_ < 0.001; Fig. 10B). These data suggest that corticosterone and high-dose insulin induce insulin resistance mainly in white adipocytes, but not in brown adipocytes.

### Effect of corticosterone treatment on gene expression in skeletal muscle

Not only adipose tissues, but also skeletal muscle is an important glucose-consuming organ that contributes to whole-body GCR, especially at the postprandial state, and GCs have been shown to reduce glucose uptake in the muscle by counteracting the effect of insulin (1, 4). We therefore investigated whether corticosterone treatment affected the gene expression profile in skeletal muscle in a sex-dependent manner. The expressions of *Nr3c1* (GR) and *Nr3c2* (MR) were not significantly different between male and female mice and were not affected by corticosterone treatment (Table 5). Corticosterone treatment significantly increased the mRNA expression of the GR target genes *Fkpb5* and *Tsc22d3* in both sexes (Table 5).

**Table 5.** mRNA Expression in Skeletal Muscle

Genes	Sig.	M Veh	M Cort	F Veh	F Cort
GR, MR, and GR target genes					
* Nr3c1*	—	1.00 ± 0.17	0.90 ± 0.06	0.88 ± 0.20	0.91 ± 0.15
* Fkbp5*	S,C,×	1.00 ± 0.10	18.11 ± 1.06^*a*^	0.69 ± 0.13	12.49 ± 2.29^*a*^^,^^*b*^
* Tsc22d3*	C	1.00 ± 0.20	3.99 ± 0.67^*a*^	1.07 ± 0.21	4.30 ± 0.95^*a*^
* Nr3c2*	—	1.00 ± 0.10	1.36 ± 0.29	1.22 ± 0.34	1.21 ± 0.13
Muscle atrophy					
* Foxo1*	C	1.00 ± 0.13	12.80 ± 2.11^*a*^	1.30 ± 0.30	14.25 ± 3.46^*a*^
* Klf15*	C	1.00 ± 0.18	2.70 ± 0.63^*a*^	1.22 ± 0.24	2.23 ± 0.36
Glucose transport					
* Irs1*	S,C,×	1.00 ± 0.17	0.66 ± 0.10	2.24 ± 0.45^*b*^	0.61 ± 0.19^*a*^
* Slc2a1*	—	1.00 ± 0.27	1.60 ± 0.61	1.08 ± 0.13	0.81 ± 0.26
* Slc2a4*	—	1.00 ± 0.08	1.04 ± 0.09	1.01 ± 0.16	1.24 ± 0.12
* Chrebpb*	C	1.00 ± 0.35	13.06 ± 1.22^*a*^	0.80 ± 0.06	18.04 ± 10.57^*a*^

Gene expression was normalized to *Gapdh* and *Rn18s* expression and expressed relative to the vehicle-treated male mice. Sig. indicates significant effects (*P* < 0.05) analyzed with two-way ANOVA.

Abbreviations: C, corticosterone treatment; S, sex; ×, interaction of corticosterone treatment and sex.

^a^ *P* < 0.05, for effect of corticosterone treatment in mice of the same sex, by *post hoc* test.

^b^ *P* < 0.05, for sex difference between mice with the same treatment, by *post hoc* test.

Another hallmark of GC excess is GC-mediated muscle atrophy by reducing muscle protein synthesis and degrading muscle proteins. Corticosterone treatment strongly upregulated the mRNA expression of the muscle atrophy-related genes *Foxo1* and *Klf15* in both male and female mice (Table 5).

Vehicle-treated female mice had a higher *Irs1* mRNA expression than did vehicle-treated male mice, but corticosterone treatment reduced *Irs1* mRNA expression to a similar level in male and female mice (Table 5). Whereas the expression of *Slc2a1* and *Slc2a4* mRNA was not significantly affected by corticosterone treatment, *Chrebpb* mRNA expression was remarkably elevated by corticosterone treatment in both sexes (Table 5).

### Effect of corticosterone treatment on gene expression in liver

We also determined hepatic gene expressions of the vehicle- and corticosterone-treated mice because the liver is the central organ regulating whole-body glucose homeostasis, especially in the fasting state when hepatic gluconeogenesis is crucial to maintain euglycemia (1, 4). *Nr3c1* mRNA expression tended to be higher in female mice than in male mice but was not significantly affected by corticosterone treatment (Table 6). However, corticosterone treatment clearly induced *Fkpb5* and *Tsc22d3* mRNA expression in both sexes (Table 6). Regarding transcriptional regulation of genes encoding gluconeogenic enzymes, corticosterone treatment upregulated the mRNA expression of *Pck1* (phosphoenolpyruvate carboxykinase 1, also known as PEPCK) and *Pcx* (pyruvate carboxylase) in a sex-independent manner (Table 6).

**Table 6.** mRNA Expression in Liver

Genes	Sig.	M Veh	M Cort	F Veh	F Cort
GR, MR, and GR target genes					
* Nr3c1*	(S)	1.00 ± 0.05	1.08 ± 0.08	1.41 ± 0.17^*a*^	1.20 ± 0.22
* Fkbp5*	C	1.00 ± 0.22	40.38 ± 7.71^*b*^	4.09 ± 0.68	31.63 ± 6.59^*b*^
* Tsc22d3*	C	1.00 ± 0.01	5.27 ± 0.57^*b*^	1.55 ± 0.25	4.50 ± 0.51^*b*^
* Nr3c2*	—	1.00 ± 0.09	1.00 ± 0.16	1.15 ± 0.13	1.22 ± 0.31
Gluconeogenesis					
* Pck1*	C	1.00 ± 0.13	2.30 ± 0.33^*b*^	1.26 ± 0.18	2.23 ± 0.23^*b*^
* Pcx*	(×),C	1.00 ± 0.05	1.92 ± 0.25^*c*^	1.03 ± 0.06	1.41 ± 0.15
Glucose transport					
* Irs1*	—	1.00 ± 0.15	0.70 ± 0.11	0.99 ± 0.09	1.02 ± 0.10
* Irs2*	×,S,C	1.00 ± 0.09	0.55 ± 0.02^*b*^	1.88 ± 0.05^*d*^	0.74 ± 0.07^*b*^
* Slc2a2*	×,C	1.00 ± 0.09	1.84 ± 0.22^*b*^	1.43 ± 0.17	1.43 ± 0.19
* Chrebpb*	(S),C	1.00 ± 0.11	1.69 ± 0.10^*b*^	0.97 ± 0.05	1.31 ± 0.18^*a*^^,^^*c*^

Gene expression was normalized to *Actb* and *Rn18s* expression and expressed relative to the vehicle-treated male mice. Sig. indicates significant effects (*P* < 0.05) analyzed with two-way ANOVA. Symbols in parentheses [*e.g.*, (S)] indicate a tendency to significance (*P* < 0.10).

Abbreviations: C, corticosterone treatment; S, sex; ×, interaction of corticosterone and sex.

^a^ *P* < 0.10 (tendency to significance), for sex difference between mice with the same treatment, by *post hoc* test.

^b^ *P* < 0.05, for effect of corticosterone treatment in mice of the same sex, by *post hoc* test.

^c^ *P* < 0.10 (tendency to significance), for effect of corticosterone treatment in mice of the same sex, by *post hoc* test.

^d^ *P* < 0.05, for sex difference between mice with the same treatment, by *post hoc* test.

Whereas *Irs1* mRNA expression was not significantly affected by sex or corticosterone treatment, *Irs2* mRNA expression was higher in vehicle-treated female mice than in male mice and was reduced by corticosterone treatment to a comparable level in both sexes (Table 6). Corticosterone treatment induced transcription of *Slc2a2* (also known as *Glut2*), a major glucose transporter for hepatic glucose uptake, in male mice only (Table 6). Furthermore, corticosterone treatment increased *Chrebpb* mRNA expression in both sexes, but this increase tended to be higher in corticosterone-treated male mice than in female mice (Table 6).

## Discussion

This study demonstrates that a high-dose corticosterone treatment causes insulin resistance in both sexes of mice, but with a more severe phenotype in male than in female mice. Our results also show that corticosterone-treated female mice displayed a more protective feature in WAT expansion and adipokine secretion than did corticosterone-treated male mice.

The 2-week treatment with high-dose corticosterone induced an insulin-resistant state more potently in males than in females, which was confirmed by high FBI concentrations, and hence an increase in HOMA-IR levels. Moreover, in-depth analyses indicated that glucose metabolism was more disturbed in male mice than in female mice. First, the corticosterone-induced increase in FBI levels slightly decreased FBG levels only in female mice. Second, corticosterone treatment elevated NFBG levels in male mice but not in female mice, resembling the early pathognomonic postprandial hyperglycemia as a sign of GC-induced diabetes mellitus in humans (44). Third, the increases in glucose and insulin levels after an IPGTT were more severe in corticosterone-treated male mice than in female mice.

To gain insight into the underlying mechanisms of the corticosterone-induced insulin resistance, especially the resemblance of a postprandial-like state, we determined EGP and GCR using an IPGTT enriched with a stable isotope tracer, that is, [U-^13^C_6_]-d-glucose. In contrast to the more severe whole-body insulin resistance in the corticosterone-treated male mice, the high blood insulin concentrations upon corticosterone treatment resulted in lower glucose production in male mice but not in female mice. Because GCs stimulate and insulin suppresses hepatic gluconeogenesis, it is hard to separate the contribution of these two factors in the control of EGP. Quinn *et al.* (45) have shown that female mice have a higher hepatic susceptibility to GCs. Thus, more pronounced actions of GCs in female mice might overrule the inhibitory effect of insulin on EGP or, alternatively, the FBG levels of corticosterone-treated mice were at their lowest limit, which requires EGP to prevent symptomatic hypoglycemia. Corticosterone treatment had opposite effects on GCR in male and female mice. GCR tended to increase in female mice but tended to decrease in male mice. These findings confirm that peripheral insulin resistance was more severe in corticosterone-treated males than in corticosterone-treated females because the elevated insulin levels by corticosterone treatment should have increased GCR substantially in both sexes. Altogether, our data show that the sex-differential effects of high-dose corticosterone treatment on insulin sensitivity were mainly driven by the more pronounced insulin resistance of peripheral tissues in male mice.

Remarkably, we observed that the BW of corticosterone-treated female mice in this study increased much more than that of corticosterone-treated male mice. This seems in contrast to the more severe insulin resistance in corticosterone-treated male mice, as weight gain usually links with an increase in insulin resistance (46, 47). The more pronounced increase in BW in female mice could be explained by the well-known effect of GC-induced muscle atrophy (48). Because lean mass of male C57BL/6J mice at this age is in general 6 g heavier than that of female mice (49), corticosterone treatment could lead to a substantial reduction in muscle mass in male mice and thereby mask an increase in fat mass, resulting in an unchanged total BW. Nevertheless, we observed that the fold increase in WAT mass was greater upon corticosterone treatment in female mice than in male mice, suggesting a sex-dependent expansion rate of WAT to GCs.

WAT stores lipid and can expand by enlarging cell size (hypertrophy), increasing cell number (hyperplasia), or a combination of both (50, 51). Hypertrophic expansion is considered more detrimental than hyperplasic expansion, as hypertrophy is associated with reduced insulin sensitivity (50). Adipose tissue expansion generally shows a depot-related pattern; that is, subcutaneous WAT is more hyperplastic than visceral WAT (51). Our finding that corticosterone-treated female mice tended to have larger WAT depots but smaller adipocytes, together with a lower mRNA expression of *Adamts1* and *Lep*, than do corticosterone-treated male mice suggests that female mice displayed more hyperplastic expansion in WATs than did male mice upon corticosterone treatment. This is in accordance with a previous study showing that corticosterone-induced adipocyte expansion was greater in ovariectomized female rats than in sham-operated female rats, which suggests a role for sex steroids in the sex difference in adipose depot expandability (52).

Besides its fundamental function as an energy reservoir, WAT secretes a number of adipokines that are essential mediators regulating systemic energy homeostasis and are also linked with the mode of expansion as previously discussed (50, 53). We found that corticosterone treatment increased *Lep* expression in gWAT, iWAT, and BAT and increased circulating leptin levels in both sexes of mice, which was linked with an increased WAT mass and was in line with other studies (54). Moreover, the increased food intake after corticosterone treatment might indicate leptin resistance in the corticosterone-treated mice (53, 54). Circulating levels of total and HMW adiponectin and the A/L ratio are considered reliable indicators for assessing adipocyte dysfunction and metabolic disorder because adiponectin levels decline in obesity and insulin resistance (41, 50, 55, 56). Also in our study, both the A/L ratio and HMW adiponectin/leptin ratio were reduced in the corticosterone-treated mice. Nevertheless, circulating levels of total adiponectin and HMW adiponectin were elevated upon corticosterone treatment, especially in female mice. In accordance with our data, a previous study in male mice showed that prednisolone treatment increased plasma adiponectin levels and the effect was more pronounced when administering prednisolone to the castrated mice (57). Expression of *Adipoq* in gWAT, iWAT, and BAT of corticosterone-treated mice, however, was lower or tended to be lower than that of the vehicle-treated mice. This contradictory effect on gene expression and total circulating protein level might be explained by the compensatory effect of insulin because our *in vitro* studies in cultured 3T3-L1 white adipocytes and T37i brown adipocytes revealed opposite effects of insulin and corticosterone on the production and secretion of adiponectin. More comprehensive *in vivo* studies are needed to elucidate this counterregulatory effect of insulin and corticosterone on adipocyte adaptations.

Note that our finding on *Adamts1* expression is different from a previous study that reported that dexamethasone-treated mice had a reduced ADAMTS1 abundance in WAT (40). However, another study demonstrated that cortisol treatment of cultured preadipocytes, obtained from WAT of healthy women who had undergone hysterectomy, upregulated *ADAMTS1* expression (58). These discrepancies may be caused by a different type, dosage, or duration of GC used among experiments. Unlike the endogenous GCs cortisol and corticosterone that can activate both GR and MR, dexamethasone is a potent GR activator with minimal mineralocorticoid effects (59), and *Adamts1* is reported to be an MR target gene, at least in cardiomyocytes (60).

Concerning the IPGTT data, an absence in spike of blood glucose levels in corticosterone-treated female mice is unlikely caused by a technical error because [U-^13^C_6_]-d-glucose can be detected in the blood of the mice and the IPGTT was performed simultaneously in all groups of mice. We speculate that this unique blood glucose pattern in the corticosterone-treated female mice reflects an effective *β*-cell response to secrete insulin for handling the injected glucose. This is in general confirmed by a recent study by Gasparini *et al.* (61) in which it was shown that only female mice remained insulin-sensitive after 4 weeks of treatment with ∼250 µg corticosterone per day in drinking water. Although insulin sensitivity was not assessed directly in our study, note that we found an increase in fasting insulin levels in both male and female mice whereas Gasparini *et al.* (61) found that corticosterone-treated female mice had an unchanged insulin concentration. This is likely due to a higher induction of systemic corticosterone levels by the high-dose corticosterone treatment in our study. Further studies using hyperinsulinemic–euglycemic clamp, the gold standard method for assessing insulin sensitivity, are needed to elucidate the effect of chronic high-dose corticosterone treatment.

Another limitation of our study is the lack of data on energy expenditure of the animals, as we think that the increased food consumption can only partially explain the enhanced fat accumulation and weight gain of the corticosterone-treated mice. Thus, performing quantitative measurement of physical activity together with indirect calorimetry is needed in future studies on sex-dependent corticosterone effects.

A few studies have shown an apparent interaction of sex steroids with GC activity. For instance, estrogen depletion in female mice resulted in a GC-driven development of hepatic steatosis (45). The consequences of this on whole-body metabolism was not addressed in the aforementioned study, but another study revealed that corticosterone-induced metabolic derangements, such as BW gain and WAT expansion, were more severe in ovariectomized than in sham-operated female rats and were counteracted by estradiol supplementation (52). Recently, androgens were also reported to modulate GR activity in liver and adipose tissue of male mice (62). Although some signs/symptoms such as muscle wasting, osteoporosis, purple striae, and nephrolithiasis are more frequent in male patients with Cushing disease, the prevalence of metabolic derangements in Cushing syndrome, such as obesity and glucose abnormalities, does not differ between men and women (63–65). In contrast, metabolic derangements upon systemic or topical use of corticosteroids appeared slightly more frequent in women than in men (66). Thus, despite the irrefutable fact that GCs have more metabolically negative effects in male rodents, which can at least partly be attributed to differences in sex steroid, the clinical manifestation is unclear and requires more dedicated studies.

In conclusion, this study illustrates a sex-dependent insulin resistance caused by continuous treatment with high-dose corticosterone. Morphological changes upon metabolic challenges and adipokine secretions from adipose tissues indicate favorable protective mechanisms in female mice. This result warrants more careful evaluation of sex as a factor in the glucose monitoring strategy of patients receiving corticosteroid therapy.

## Data Availability

The datasets generated during and/or analyzed during the current study are not publicly available but are available from the corresponding author on reasonable request.

## Abbreviations:

A/L: adiponectin/leptin
AUC: area under the curve
aWAT: anterior subcutaneous WAT
BAT: brown adipose tissue
BW: body weight
ChREBP: carbohydrate-responsive element-binding protein
EGP: endogenous glucose production
FBG: fasting blood glucose
FBI: fasting blood insulin
FBS: fetal bovine serum
FF-BSA: fatty acid–free BSA
GC: glucocorticoid
GC-MS: gas chromatography–mass spectrometry
GCR: glucose clearance rate
GR: GC receptor
gWAT: gonadal WAT
HOMA-IR: homeostatic model assessment of insulin resistance
HMW: high-molecular-weight
HPA: hypothalamic-pituitary-adrenal
IPGTT: IP glucose tolerance test
iWAT: inguinal WAT
MR: mineralocorticoid receptor
NFBG: nonfasting blood glucose
*P*_C_: *P* value for corticosterone treatment
PFA: paraformaldehyde
*P*_I_: *P* value for insulin treatment
*P*_PC_: *P* value for pretreatment with corticosterone
*P*_PI_: *P* value for pretreatment with insulin
*P*_S_: *P* value for sex
P/S: penicillin/streptomycin
*P*_SI_: *P* value for stimulatory insulin
*P*_T_: *P* value for time (duration) of treatment
RT: room temperature
WAT: white adipose tissue
